# Interspecific and interploidal gene flow in Central European *Arabidopsis *(Brassicaceae)

**DOI:** 10.1186/1471-2148-11-346

**Published:** 2011-11-29

**Authors:** Marte H Jørgensen, Dorothee Ehrich, Roswitha Schmickl, Marcus A Koch, Anne K Brysting

**Affiliations:** 1 Centre for Ecological and Evolutionary Synthesis (CEES), Department of Biology, University of Oslo, P.O. Box 1066 Blindern, NO-0316 Oslo, Norway; 2 Institute for Arctic and Marine Biology, University of Tromsø, NO-9037 Tromsø, Norway; 3 Centre for Organismal Studies (COS) Heidelberg, Department of Biodiversity and Plant Systematics, University of Heidelberg, Im Neuenheimer Feld 345, D-69120 Heidelberg, Germany

## Abstract

**Background:**

Effects of polyploidisation on gene flow between natural populations are little known. Central European diploid and tetraploid populations of *Arabidopsis arenosa *and *A. lyrata *are here used to study interspecific and interploidal gene flow, using a combination of nuclear and plastid markers.

**Results:**

Ploidal levels were confirmed by flow cytometry. Network analyses clearly separated diploids according to species. Tetraploids and diploids were highly intermingled within species, and some tetraploids intermingled with the other species, as well. Isolation with migration analyses suggested interspecific introgression from tetraploid *A. arenosa *to tetraploid *A. lyrata *and vice versa, and some interploidal gene flow, which was unidirectional from diploid to tetraploid in *A. arenosa *and bidirectional in *A. lyrata*.

**Conclusions:**

Interspecific genetic isolation at diploid level combined with introgression at tetraploid level indicates that polyploidy may buffer against negative consequences of interspecific hybridisation. The role of introgression in polyploid systems may, however, differ between plant species, and even within the small genus *Arabidopsis*, we find very different evolutionary fates when it comes to introgression.

## Background

Polyploidy, i.e. whole genome duplication, has long been considered a major evolutionary force in the Plant Kingdom [see e.g., [1-5]], and even though large advances in our understanding of polyploidy have been made during the last couple of decades, there are still many questions unanswered [reviewed by [6]]. We still don't have a general agreement on classification of polyploids, for instance. Some authors work with strict taxonomic definitions; autopolyploids are the result of polyploidisation events involving only a single species, and allopolyploids are the result of interspecific hybridisation [e.g., [3]]. Others base their definitions on inheritance patterns and the presence or absence of multivalents [e.g., [7]]. However, most would agree that auto- and allopolyploids are the extremes of a continuous range. There are also still controversies about how polyploids should be treated taxonomically. Soltis et al. [8] suggest that autopolyploids deserve species rank taxonomically, with ploidal level as part of the name. Others do not even give allopolyploids species status due to lack of morphological distinctness (e.g., lack of diagnostic qualitative and discrete characters), and include them as subspecies of one of the parents [e.g., [9]]. Yet others separate morphological and biological species where the first may contain several of the latter [e.g., [10]].

Traditionally, polyploidisation events have been considered to result in total reproductive isolation of the new polyploid from the parent (s), and thus regarded as instant speciation [e.g., [11]]. More recent research has shown that recurrent formation of polyploids and triploid bridges contribute to interploidal gene flow [3,12,13]. To what extent, however, is still not known [6]. Multiple independent polyploidisation events have been shown to be common for both allopolyploids [e.g., [14,15]] and autopolyploids [e.g., [16-18]]. Population studies and modelling of sympatric *Chamerion angustifolium *(L.) Holub revealed that autotetraploids are not necessarily instantly isolated from their diploid progenitors, but that the isolation can become more prevalent through time [19,20]. Slotte et al. [21] showed that there is unidirectional gene flow from diploid *Capsella rubella *Reuter to its allotetraploid descendant *C. bursa-pastoris *(L.) Medicus. Furthermore, if polyploidisation events result in immediate isolation from the progenitors, the result should be a major bottleneck. However, several studies have shown higher genetic diversity in polyploids compared to their progenitors [22-26], although this is not always the case [14,27-30]. The increased diversity may be the result of either recurrent formation of the polyploids [e.g., [1]], or past or ongoing interploidal gene flow through, for instance, triploid bridges [e.g., [19]]. These different models of polyploidisation can be seen as a gradient. Single event polyploidisation with subsequent reproductive isolation represents one end of this gradient whereas polyploidisation with ongoing gene flow or recurrent polyploidisation represents the other. Instances where polyploidisation is followed by historical gene flow which later stopped, or where polyploidisation is followed by reproductive isolation and subsequent gene flow in the form of secondary contact [31], could be considered as intermediate forms. Criteria and methods to distinguish between these different categories, though, have not yet been proposed [6].

*Arabidopsis *(DC.) Heynh. is a small genus consisting mostly of diploids, but includes both allopolyploids [14,32-35] and taxonomic autopolyploids [36-41]. As the genus includes the geneticists' pet plant *A. thaliana *L., plenty of molecular tools are available also for its relatives [e.g., the recent release of the *A. lyrata *genome, [42]], making the genus ideal for studying polyploid evolution. In Central Europe two species have been recorded with two ploidal levels each: *A. arenosa *(L.) Lawalrée (hereafter *arenosa*) represents a complex species aggregate [38] with diploid taxa occurring mainly in the Carpathians and possibly in a few regions further south in Hungary and Croatia, whereas tetraploid *arenosa *is found in most of Central Europe. In contrast *A. lyrata *(L.) O'Kane & Al-Shehbaz (hereafter *lyrata*) is mostly diploid throughout its European distribution range, but several tetraploid populations are found in the Eastern Austrian Forealps and neighbouring regions [39,43,44]. Schmickl and Koch [45] detected significant levels of introgression from tetraploid *arenosa *into the gene pool of diploid and tetraploid *lyrata*, resulting in introgressed tetraploid populations in the Eastern Austrian Forealps and the northerly adjacent Danube Valley, using microsatellite markers and morphometrics. These results, based on genetic and morphological admixture and differentiation, suggest past and ongoing gene flow between the two taxa. To distinguish between ancient and recent gene flow, especially in polyploid complexes, has long been controversial, and no clear criteria have yet been commonly acknowledged [6]. Here we use low-copy nuclear and plastid DNA sequences from the *Arabidopsis *model system and different analytical methods to study interspecific and interploidal gene flow in *lyrata *and *arenosa*, specifically asking, 1) whether interploidal gene flow takes place in one or both directions, 2) how polyploidisation affects interspecific introgression and 3) whether it is possible to distinguish between recurrent formation and introgression from progenitors.

## Methods

### Material

**Figure 1:**
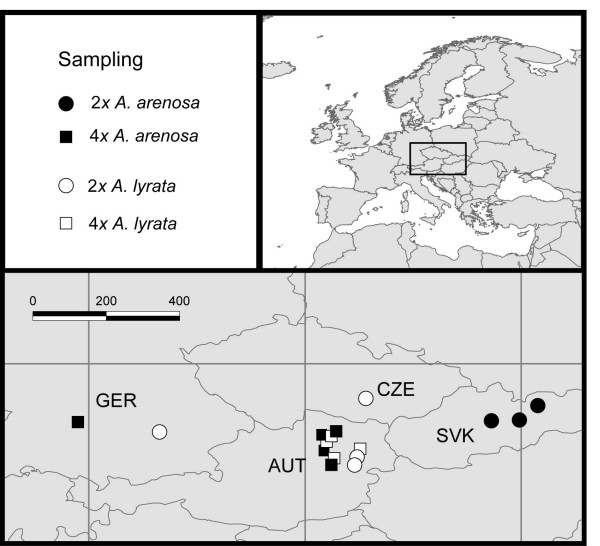
**Central European *Arabidopsis *populations included in this study**. Taxon is given by colour: *A. arenosa *- black and *A. lyrata *- white. Ploidal levels are given as circle - diploid and square - tetraploid. Country names are abbreviated: AUT - Austria, CZE - the Czech Republic, GER - Germany, and SVK - Slovakia.

Three to five Central European populations of each ploidal level (2*x *and 4*x*) from both *arenosa *and *lyrata *were included in this study (Table 1; Figure 1) with a total of 16 populations. *Lyrata *2*x*, *lyrata *4*x *and *arenosa *4*x *were mostly sampled in close proximity in the Eastern Austrian Forealps. *Arenosa *2*x *was sampled in Slovakian Carpathians. The material was collected in the wild as living plants or seeds from defined single mother plants and grown in the Botanical Garden, University of Heidelberg, and the Phytotron, University of Oslo. Fresh leaves of 1-43 plants from most populations (274 individuals in total; Table 1) were later collected for flow cytometry analyses. Leaves from two specimens per population were dried using silica gel to preserve DNA before extraction.

**Table 1:** Sampling of *Arabidopsis arenosa *and *A. lyrata *included in this study.

Taxon	Ploidal level	**Population**^**1**^	**Locality**^**2**^	**# FC**^**3**^
*arenosa*	2*x*	a2_SVK1	SVK: Vysoké Tatry; Prešovský kraj; Belianske Tatry; Zadné Med'odoly Valley; Kopské Sedlo (131)	5
		a2_SVK2	SVK: Nízke Tatry Mts.; Pusté Pole (915140)	30
		a2_SVK3	SVK: Vel'ká Fatra Mts.; Harmanec; Malý Šturec Sedlo (915141)	33
	4*x*	a4_GER	GER: Southern Germany; Swabian Alps; Wental; Felsenmeer (123)	-
	^4^	a4_AUT1	AUT: Lower Austria; Eastern Alps; SSW St. Aegyd am Neuwalde; Kernhof; rocky batter next to street opposite depot (81-915142)	26
		a4_AUT2	AUT: Lower Austria; Waldviertel; Wachau; NNE Weißenkirchen; Achleiten (3)	12
		a4_AUT3	AUT: Lower Austria; Kamp Valley; S Stiefern; parking site with view on railway bridge (89)	-
		a4_AUT4	AUT: Lower Austria; Waldviertel; Wachau; forest road from Scheibenbach towards Pfaffental (20)	4
*lyrata*	2*x*	l2_GER	GER: Bavaria; Veldenstein Forest; street from Velden to Pfaffenhofen (112)	17
	^5^	l2_CZE	CZE: SW Brno; NW Ivanice; between Nova Ves and Oslavany; slope above Oslava River (96)	9
		l2_AUT1	AUT: Lower Austria; street from Pernitz to Pottenstein (88-915143)	43
		l2_AUT2	AUT: Lower Austria; S Vienna; Bad Vöslau; rocks near Vöslauer Hütte (74-915145)	28
	4*x*	l4_AUT1	AUT: Lower Austria; Waldviertel; Wachau; E Dürnstein; small hill N Franzosendenkmal (13)	24
		l4_AUT2	AUT: Lower Austria; S Vienna; Mödling; Castle ruin Mödling (66-915144)	21
		l4_AUT3	AUT: Lower Austria; Dunkelstein Forest; Wachau; N Bacharnsdorf (50)	21
		l4_AUT4	AUT: Lower Austria; Schrambach between Freiland and Lilienfeld (116)	1

^1^Two individuals from each population were sequenced.

^2^Country names are abbreviated as follows: AUT - Austria, CZE - Czech Republic, GER - Germany, and SVK - Slovakia. Brackets following localities give original collection number given in Schmickl and Koch [45].

^3^# FC gives the number of individuals analysed with flow cytometry. The populations a4_GER and a4_AUT3 were not included in the flow cytometry analysis, but multi-allelic microsatellite loci suggest they are tetraploid (Schmickl and Koch, unpublished).

^4^a4_AUT1 contained a single diploid individual, the others were tetraploid.

^5^l2_CZE contained a single triploid individual, the others were diploid.

### Flow cytometry

Relative nuclear DNA content of 274 specimens from 14 populations (Table 1) were estimated by flow cytometry analyses performed by G. Geenen (Plant Cytometry Services, Schijndel), using DAPI staining, the *Arabidopsis *buffer described in Doležel and Suda [46] and *Ilex crenata *Thunb. 'Fastigiata' as internal standard, otherwise following the protocol described in Jørgensen *et al*. [30]. The populations a4_GER and a4_AUT3 were not included in the analyses as we did not have living material from these at the time of the analysis. However, microsatellite data for these populations indicate that they are tetraploid (Schmickl and Koch, in preparation). T-tests were done in SPSS 16.0 (SPSS Inc., Chicago) to test for differences in means of nuclear DNA content between the taxa.

### DNA extraction, cloning, and sequencing

Whole genomic DNA was extracted from leaf tissue using the DNeasy Plant Mini protocol (Qiagen, Hilden). Polymerase chain reaction (PCR) of the low-copy nuclear regions chalcone synthase (CHS) and short chain alcohol dehydrogenase (*sc*ADH) was carried out in 25 μl volumes with 1× DyNAzyme EXT buffer (Finnzymes, Espoo), 0.2 mM of each dNTP, 0.6 μM of each primer (Additional file 1, Table S1), 0.2 U DyNAzyme EXT DNA polymerase (Finnzymes), and 2 μl 10 times diluted DNA template. Thermocycling conditions consisted of 3 min at 94°C, and 35 cycles of 30 s at 93°C, 30 s at 55°C, 2.5-3 min at 70°C, and a final extension for 5 min at 70°C. PCR products were cloned using the TOPO-TA kit for sequencing with the pCR4-TOPO vector (Invitrogen, Carlsbad). Colonies were checked for inserts by running a PCR with M13 or T7 primers. At least six insert-containing clones from each PCR reaction were sequenced in both directions. The plastid region *trn*L-F was amplified using PuReTaq Ready-To-Go PCR beads (GE Healthcare, Waukesha) with 0.6 μM of each primer (Additional file 1, Table S1) and 2 μL 10 times diluted DNA template. For each DNA region, both strands were sequenced using BigDye v3.1 7 cycle sequencing kit (Applied Biosystems, Foster City) and M13F/M13R or T7 primers. Products of the cycle-sequencing reactions were separated on an ABI 3700 Genetic Analyzer (Applied Biosystems). The resulting sequences were assembled and edited using Vector NTI advance 10 (Invitrogen), and consensus sequences representing each allele and alignments were made using BioEdit version 7.0.5 [47]. For the plastid *trn*L-F region, only the first 700 base pairs were included in the analyses, to avoid unambiguous alignment due to the presence of pseudogenes [48,49].

PCR-mediated recombinants (chimeras) constitute a well-known problem in PCR-based cloning protocols [e.g. [50-55]], and to distinguish between PCR-mediated and real recombinants is not possible via PCR-based methods. However, the risk of obtaining recombinants as PCR artefacts increases with the concentration of template [51], and the expected frequency of these should be lower than for real recombinants [50,52]. In this study we omitted clones that were recombinants of other cloned sequences from the same individual, and that were present at low frequencies, as PCR artefacts. Discrepant bases supported by only a single clone were assumed to be due to polymerase reading error and were corrected based on consensus sequences of other clones from the same individual.

### Data analyses

Intragenic recombination events may be relatively common [56], and should be taken into consideration when choosing methods for phylogenetic analysis [57]. Minimum number of recombination events [RM; [58]] per region was calculated using DnaSP version 5.10 [59]. As we found substantial recombination for most regions (Additional file 1, Table S1), phylogenetic relationships were analysed for each region using neighbour networks [60], with Jukes-Cantor distances in the program SplitsTree4 [61]. Gaps were included following the simple coding strategy introduced by Simmons and Ochoterena [62] as implemented in the software SeqState version 1.4.1 [63]. For all marker systems the datasets were analysed by: 1) splitting the individuals into subsets according to taxa, 2) including all individuals, and 3) including only diploids.

When two or three different alleles are found in a tetraploid individual, it is not possible to determine the true genotype although dosage may give an indication. Computational methods based among others on the EM algorithm have been developed to infer genotypes assuming random mating and populations at equilibrium [e.g. [64]]. As we have sampled two individuals from several distinct populations, we cannot assume equilibrium, and chose not to use any statistical method. To roughly assess the impact of assuming different numbers of allele copies, we reconstituted genotypes at random using the following approach: For each tetraploid individual with two or three alleles, a random number between 1 and 3 was generated. For individuals with two alleles, 1 corresponded to three copies of the first allele and one copy of the second (the order was arbitrary), 2 corresponded to two copies of each allele and 3 corresponded to one copy of the first allele and three copies of the second. For individuals with three alleles, 1 corresponded to duplicating the first allele, 2 to duplicating the second allele and 3 to duplicating the third allele. Three different datasets (D1, D2, D3) were generated using this approach. Assuming that the three allele proportions 1:3, 2:2, and 3:1 are equally probable for tetraploids with two distinct alleles at a locus leads to a deficit of 2:2 individuals compared to equilibrium expectations. Therefore we created a fourth dataset (D22) where all individuals with two distinct alleles were considered to have a 2:2 genotype. The four datasets were used both in diversity calculations and isolation with migration (IM) analyses.

Diversity indices were calculated for each molecular region and each species/ploidal level, separately. For the tetraploids we calculated the indices for the duplicated datasets D1, D2, D3, and D22, and averaged the estimates. Arlequin version 3.11 [65] was used to calculate gene diversity (Hd), nucleotide diversity averaged over loci (π), and average number of nucleotide differences (k) with standard deviations. Differences in diversity between ploidy levels and between taxa were assessed by two sided T-tests computed using the standard deviations given by Arlequin.

The parameters of the IM model were estimated as implemented in the program IMa2 [66,67] to assess the importance and direction of gene flow between the ploidal levels and species. Samples of *arenosa *and *lyrata *were first analysed separately, to determine whether there is gene flow between ploidal levels and in which direction it occurs. Second, in order to assess gene flow between species, both taxa with both ploidal levels were analysed together in an analysis with four populations. We assumed one ancestral population for each species, and one ancestral population for the whole complex. In such an analysis with four populations a large number of parameters have to be estimated, requiring a large amount of data to obtain reliable estimates. As the three loci available here were somewhat limited in that respect, we also analysed only the two diploid taxa to assess evidence for interspecific gene flow. All analyses involving tetraploids were carried out for the four different datasets of tetraploid genotypes. One of the assumptions of the IM model is that there is no important gene exchange with populations not included in the analysis. As there was evidence for significant gene flow between ploidal levels in both species, we did not analyse the two tetraploids together. Another assumption of IMa2 is that there is no recombination. Tests for recombination [58,68] showed, however, that there was considerable recombination in the nuclear sequences used here. The program IMgc [69] was used to find the largest subsets of the data matrix without signs of recombination (nonrecombining blocks) by removing either sequences or variable sites. The program can prioritise the number of sequences kept or the number of variable sites. We first used the default value 1 for the prioritising parameter. As some of the sequences got very short (datasets def; Additional file 2, Table S2), we produced additional subsets using a value of 0.5, retaining more variable sites and fewer sequences (datasets seq). This option was used for *arenosa *and *lyrata*, but it could not be used for the total and diploid datasets, because it reduced the number of sequences of diploid *arenosa *to three or less (Table S2). Reducing the data to non-recombining blocks reduces the amount of data and leads to a loss of diversity, which may lead to a downward bias in estimates of effective population sizes obtained from IM. Divergence time and gene flow estimates have, however, been shown to be largely unaffected [70].

The parameter estimates provided by IMa2 are scaled by the mutation rate. In order to convert them to demographic estimates, a mutation rate needs to be assumed. We followed the procedure of Slotte et al. [21]: we assumed a substitution rate of 6.5·10^-9 ^[71,72] as a lower boundary for the mutation rate and the synonymous substitution rate of 1.5·10^-8 ^per site per generation [73] as an upper boundary, and used the mean of these two estimates to calculate per-fragment mutation rates (Additional file 3, Table S3).

IMa2 uses a Bayesian approach and Markov Chain Monte Carlo (MCMC) simulations to estimate parameters. Priors for effective population sizes (q = 4N_e_*μ, where μ is the migration rate per fragment, not per bp), time since divergence (t = time*μ) and migration rates (m = migration rate/μ) were initially chosen as recommended in the IM manual [74] and adjusted according to the results of preliminary analyses. For the final runs the following values were used (all values are scaled by μ = 1.21·10^-5^): q = 17, t = 10 and m = 5 for *arenosa*, q = 20, t = 7 and m = 5 for *lyrata*, q = 15, t = 10 and m = 5 for the total dataset, and q = 12, t = 10 and m = 2 for the diploid dataset. The number of chains and the heating scheme were also tested in several preliminary runs. For the final runs we used 20 chains and heating parameters of ha = 0.96 and hb = 0.9 for analyses with two populations and 80 chains, and ha = 0.999 and hb = 0.3 for analyses with four populations. The length of the burnin was 1 000 000 MCMC iterations and estimates were based on between 10 and 27 million iterations. Mixing was assessed by trend plots for estimates over the runs and by effective sample size (ESS) values. Convergence was assessed by repeating runs several times with different random seeds. Some IMa2 runs were performed on BioHPC, Computational Biology Service Unit, Cornell University.

## Results

### DNA content

The ploidal levels for the 14 populations examined are given in Table 1. Only two populations showed signs of more than one ploidal level. The tetraploid *arenosa *population a4_AUT1 from Kernhof in Austria included one diploid individual, and the diploid *lyrata *population l2_CZE from NW Ivanice in the Czech Republic contained one triploid. The T-test showed that the two taxa had significantly different DNA content within ploidal levels for both diploids and tetraploids, *lyrata *having a slightly larger nuclear DNA content than *arenosa *in both cases (0.23 vs. 0.20, and 0.44 vs. 0.43, *P *< 0.001).

### Sequence variation and diversity

The obtained sequences were deposited to GenBank with accession numbers GQ386471-GQ386654; 75 sequences of CHS, 59 sequences of *sc*ADH, and 32 sequences of *trn*L-F (Additional file 1, Table S1). Substantial recombination has taken place for both low-copy nuclear regions. Minimum number of recombination events was 16 for CHS and 24 for *sc*ADH (Additional file 1, Table S1). For the plastid *trn*L-F region, only a single recombination event was detected.

**Figure 2:**
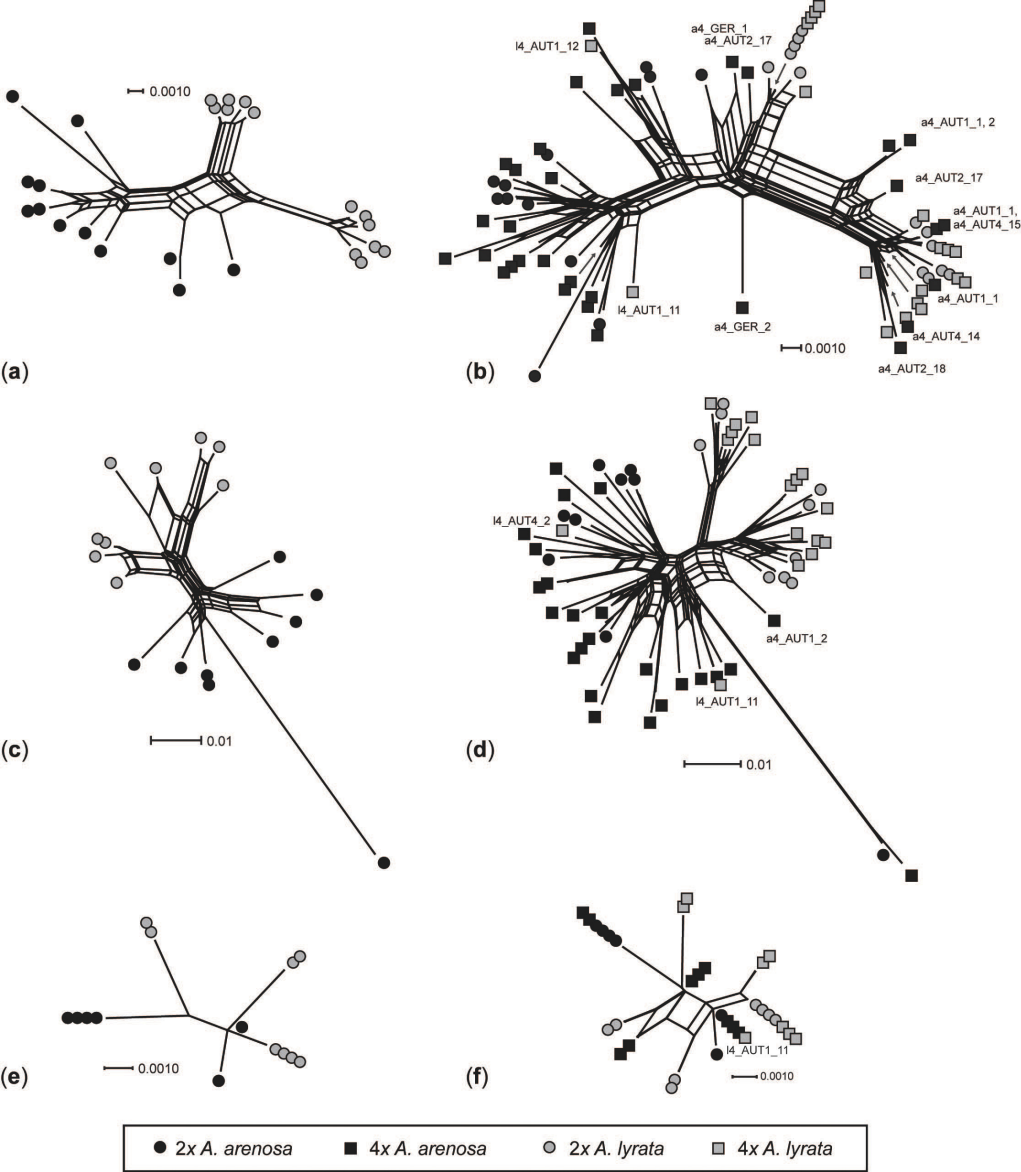
**Neighbour network analyses of diploid only (a, c, e) and both diploid and tetraploid (b, d, f) Central European *Arabidopsis arenosa *and *A. lyrata *based on (a, b) the low-copy nuclear CHS region; (c, d) the low-copy nuclear *sc*ADH region; and (e, f) the plastid region *trn*L-F**. Only specimens deviating from the majority within each taxon are named. Taxon is given by colour: *A. arenosa *- black and *A. lyrata *- grey. Ploidal levels are given as circle - diploid and square - tetraploid.

When analysed alone, the diploids were separated into two groups corresponding to named taxa in the neighbour networks based on the nuclear markers (Figure 2a, c), and partly also the plastid region (Figure 2e). CHS split the *lyrata *diploids into two distinct groups with absolutely no geographical structure; both clusters included specimens from Germany, the Czech Republic, and Austria (Figure 2a). There was no apparent geographical structure among *arenosa *specimens either. The analysis of *sc*ADH gave no additional information (Figure 2d). The two taxa didn't share *trn*L-F haplotypes, but all three *lyrata *haplotypes clustered closer to *arenosa *than to each other (Figure 2e).

Adding the tetraploids to the neighbour networks complicated the picture (Figure 2b, d, f). The majority of the tetraploids clustered according to taxa; tetraploid *lyrata *clustered with diploid *lyrata*, and tetraploid *arenosa *with diploid *arenosa*. There were, however, exceptions for all the marker systems. The CHS network grouped five tetraploid *lyrata *sequences with tetraploid *arenosa *(Figure 2b; Additional file 4, Table S4). These represent three specimens (with a mixture of *lyrata*- and *arenosa*-like alleles) of which two are from the same population (l4_AUT1), collected in Wachau, and the last one from Schrambach, also in Lower Austria (population l4_AUT4). In the *sc*ADH network, one of the specimens from the Wachau population (l4_AUT1_11) shared an allele with a tetraploid *arenosa *collected just a few kilometres away (a4_AUT2_18), whereas the specimen from the Schrambach population (14_AUT4_2) clustered with a tetraploid *arenosa *from Wachau (a4_AUT4_15; Figure 2d; Additional file 4, Table S4).

In the CHS network ten tetraploid *arenosa *sequences clustered with the *lyrata *groups (Figure 2b). These represent seven specimens (with a mixture of *lyrata*- and *arenosa*-like alleles; Additional file 4, Table S4), most of them from Lower Austria (populations a4_AUT1, a4_AUT2, and a4_AUT4), but a single one from Germany (a4_GER). Only one of these tetraploid *arenosa *specimens (a4_AUT1_2) contained a *lyrata*-like *sc*ADH allele and clustered with *lyrata *in the network (Figure 2d; Additional file 4, Table S4).

The plastid *trn*L-F network separated specimens according to taxa with one exception: the same tetraploid *lyrata *specimen from Wachau (l4_AUT1_11), which clustered with *arenosa *also in the CHS and *sc*ADH networks, shared a haplotype with diploid and tetraploid *arenosa *(a2_SVK2, a4_AUT2, and a4_GER, Figure 2f).

To summarise, these networks basically told the same story with major splits between *lyrata *and *arenosa*, and with ploidal levels to a high degree intermingled within each taxon. Deviations from this pattern were found more or less in the same populations for the different markers; tetraploid *arenosa*: a4_AUT1, a4_AUT2, a4_AUT4 and tetraploid *lyrata*: l4_AUT1 and l4_AUT4, all populations from Lower Austria where the two taxa are sympatric (Table 1; Additional file 4, Table S4).

**Figure 3:**
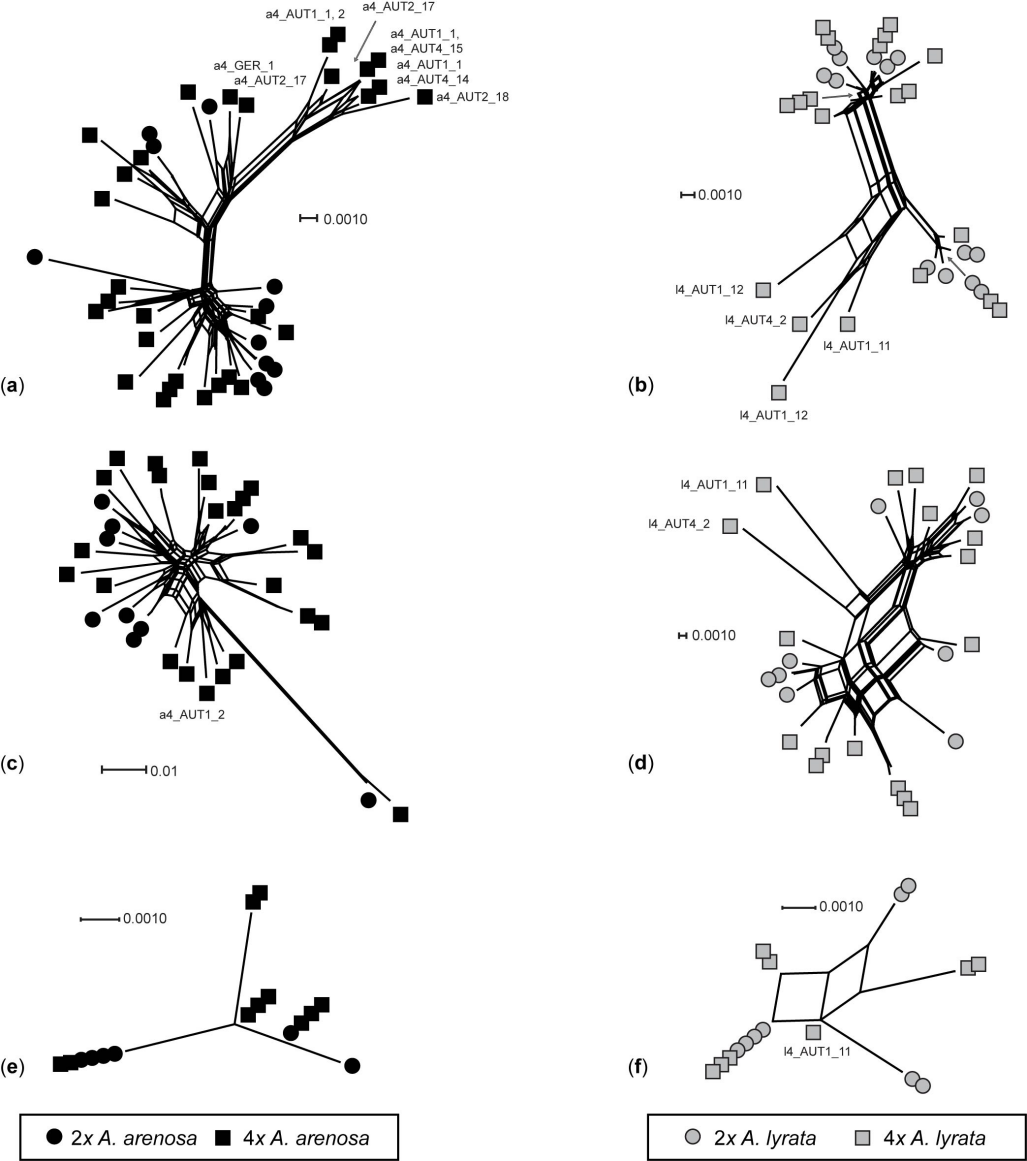
**Neighbour network analyses of diploid and tetraploid Central European *Arabidopsis arenosa *(a, c, e) and *A. lyrata *(b, d, f) species. (a, b) are based on the low-copy nuclear CHS region; (c, d) are based on the low-copy nuclear *sc*ADH region; and (e, f) are based on the plastid region *trn*L-F**. Only specimens identified as deviating from the majority within each taxon in Figure 2 are named. Taxon is given by colour: *A. arenosa *- black and *A. lyrata *- grey. Ploidal levels are given as circle - diploid and square - tetraploid.

Analysing the taxa separately for all marker systems showed that the specimens did not cluster according to ploidal level (Figure 3). For both species, the CHS and *sc*ADH networks separated groups of a few tetraploids from the remaining specimens, corresponding to the deviations mentioned above (Figure 3a, b). Otherwise specimens of different ploidal levels are completely intermingled.

**Figure 4:**
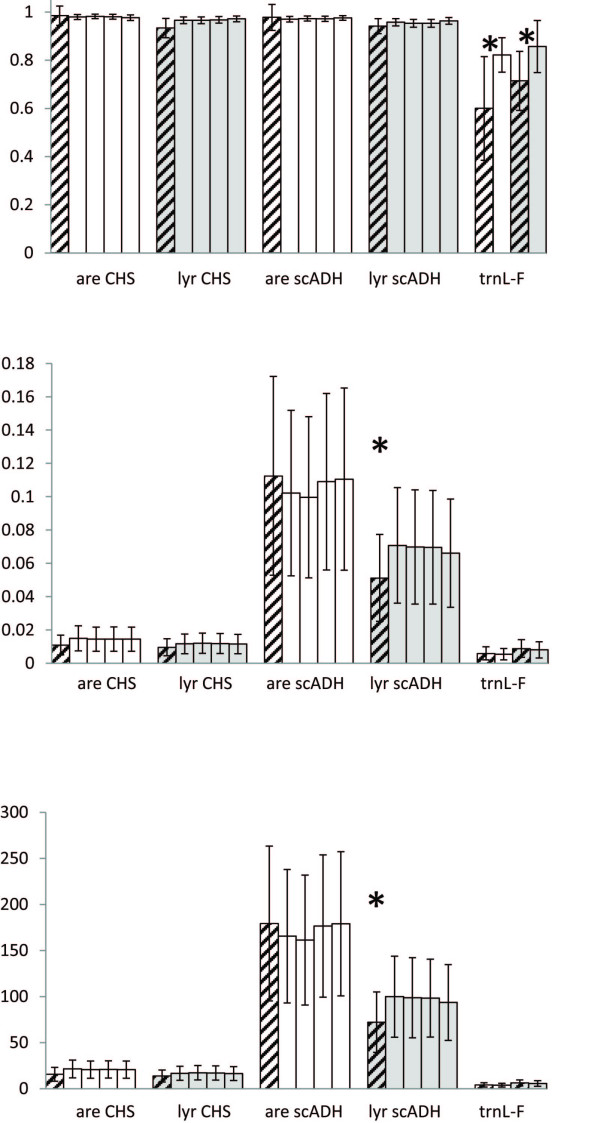
**Diversity analyses of Central European *Arabidopsis arenosa *(white) and *A. lyrata *(grey)**. Diploids are hatched, tetraploids are blank. Bars give standard deviation, * gives significance. (a) Gene diversity, Ĥ; (b) Nucleotide diversity, π; and (c) Average number of nucleotide differences, k.

Both nuclear regions showed high levels of gene diversity (Hd = 0.93-0.99; Figure 4, Additional file 5, Table S5). Differences in diversity among the duplicated tetraploid datasets (D1-22) were negligible, and there were no clear differences between taxa or ploidal levels. For *trn*L-F, the diversity was somewhat lower (Hd = 0.60-0.86), particularly for *arenosa*. For this marker the diversity was significantly higher in tetraploids than in diploids of both species (*p *< 0.05; Figure 4). There were no significant differences in nucleotide diversity (π) and average number of nucleotide differences (k) between ploidal levels or species for *trn*L-F and CHS. For scADH, however, both estimates of molecular diversity were significantly higher for *arenosa *than for *lyrata*, and this was the case for diploids and tetraploids (π: *p *< 0.005 for tetraploids and *p *< 0.02 for diploids, and k: *p *< 0.001 for tetraploids and *p *< 0.005 for diploids; Figure 4, Additional file 5, Table S5).

### Isolation with migration results

For analyses of pairs of populations all runs reached ESS values > 1000 and mixing seemed good based on trend plots. Repeated runs indicated good convergence. The analyses with four populations did, however, not perform equally well and ESS values remained < 50 for several parameters. We will therefore in the first place base our conclusions on the pair wise runs, and only mention the results of the four population runs as indicative.

**Figure 5:**
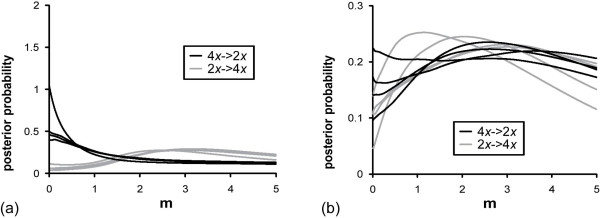
**Isolation with migration (IM) analyses of the datasets D1-D22 (see text for details): migration rates**. (a) Migration from diploid to tetraploid (grey) and from tetraploid to diploid (black) *A. arenosa*. (b) Migration from diploid to tetraploid (grey) and from tetraploid to diploid (black) *A. lyrata*.

The main aim of this study was to assess evidence for gene flow between ploidal levels and species. For *arenosa*, the IM analysis revealed strong support for gene flow from diploids to tetraploids, but not in the other direction (Figure 5, Additional files 6, 7, Fig. S1, S2). The 95% highest posterior density intervals (HPD) for the migration rate from diploids to tetraploids excluded 0 for seven of eight datasets where it was estimated reliably (four variants of tetraploid genotypes × two options of largest non-recombinant blocks; Additional file 2, Table S2), whereas the estimate of m was at the lowest value for migration from tetraploids to diploids in all cases. Estimates of the number of migrants from diploids to tetraploids were between 2.1 and 4.1, but HPD intervals were large and overlapped considerably among both migration directions (Additional file 2, Table S2). The posterior distribution for time since divergence did not go down towards 0 at the upper limit of the prior interval, independent of prior choice, and divergence time could thus not be properly estimated. Effective population size estimates were 1.5 to 2 times higher for tetraploids than for diploids (Additional file 6, Fig. S1), but as these are estimates of the effective number of genes, the estimated number of tetraploid individuals was in fact somewhat lower than for diploids.

For *lyrata*, there was also clear support for gene flow between the ploidal levels. HPD intervals for gene flow from diploids to tetraploids excluded 0 for five of eight datasets (Additional file 2, Table S2) and all estimates of m were larger than 0. For gene flow from tetraploids to diploids, HPD distributions did not reach low levels at high values for gene flow from tetraploids to diploids, making them unreliable. Still the HPD intervals excluded 0 for three out of four def datasets, and estimates of m were at the lowest point of the distribution only for one def dataset and two seq datasets, indicating gene flow from tetraploids to diploids. The discrepancies between the different datasets resulted from the fact that different parts of the sequences were kept by IMgc (Additional file 2, Table S2). The effective number of migrants per generation was estimated as 1.5 to 1.8 from diploids to tetraploids and as 1.2 to 1.4 from tetraploids to diploids. As for *arenosa*, the posterior distributions for time since divergence were not unimodal and did not provide any reliable estimates. Estimates of effective population sizes varied also somewhat between datasets and were in general not different between ploidal levels.

The results of the analysis including all four taxonomic/cytogenetic entities (Additional file 2, Table S2) were largely consistent with the results of the pair wise runs, although these estimates have to be considered unreliable due to poor performance of the MCMC (and are therefore not shown). In addition, these runs suggested gene flow between species, from diploid and tetraploid *arenosa *into tetraploid *lyrata*, and for one dataset also from diploid *lyrata *into tetraploid *arenosa*.

Analysing the two diploid species together (Additional file 2, Table S2) showed that there was no evidence what so ever for gene flow between them.

## Discussion

The overall picture indicated by our results is the following: Both the neighbour nets and the IM analysis show that there is no gene flow between the two diploid taxa, a result which is in agreement with expectations based on their presently well separated distribution areas [38]. There is, however, evidence for gene flow from diploids to tetraploids in *arenosa *and possibly for interploidal gene flow in both directions in *lyrata*. This is consistent with the intermingling of sequences of both ploidal levels revealed by the networks. The possibility of gene flow from tetraploids to diploids in *lyrata *is further supported by the triploid individual found in population l2_CZE. However, spontaneous occurrence of triploids in diploid populations is a common phenomenon and might be alternatively explained by low-frequency production of unreduced gametes. The networks including both species and ploidal levels clearly indicate mixing of lineages between species. As there seems to be no gene flow between diploids, we assume that this mixing results from gene flow into tetraploids. Consistent with this assumption, the IMa2 analysis of all four taxonomic/cytogenetic entities suggests gene flow from diploid and tetraploid *arenosa *into tetraploid *lyrata*, and possibly, with much lower frequency, also from diploid *lyrata *into tetraploid *arenosa*.

### Interspecific gene flow

It has been suggested that genotypes of tetraploids are buffered against the shock of absorbing foreign genomes, and that extensive introgression often takes place at the tetraploid level between species that are isolated from each other at the diploid level [75,76]. Our analyses of *Arabidopsis *in Central Europe show that *arenosa *and *lyrata *are good biological species at the diploid level. The network analyses show no sharing of alleles, and the main splits are between the two taxa, which is in agreement with a comprehensive large-scale analysis of the genus [77]. Furthermore, isolation with migration (IM) analyses of diploids show no gene flow from 2*x arenosa *to *lyrata *or the other way around.

We do, however, find signs of introgression in the tetraploids on both sides; several tetraploid *lyrata *sequences cluster with *arenosa *in the network analyses (especially for the CHS region, but also for the other two regions), and several tetraploid *arenosa *sequences cluster with *lyrata*.

The pattern of gene flow that we observed could alternatively be explained by the fact that diploids of the two species are spatially separated, whereas the tetraploids largely overlap in distribution range. Because we lack proper population samples in the present study, we could not formally test to what extent a correlation between genetic and geographic distance could explain our results (using for instance a Mantel test). However, the pattern resulting from the IM analyses cannot fully be explained by geography. IM indeed indicated unidirectional gene flow into tetraploids in *arenosa*, despite ploidal levels being at present allopatric. Gene flow from diploid *arenosa *into tertraploid *lyrata *was also suggested by some of the IM results. Therefore our results are very likely to reflect more than simple isolation by distance.

To our knowledge there are not many studies that have dealt with gene flow between sister species that contain two (or more) ploidal levels. Luttikhuizen *et al*. [23] found higher genetic diversity in autotetraploid *Rorippa amphibia *(L.) Besser compared to conspecific diploids using microsatellites, and suggested that introgression as well as multiple origins of the tetraploids might have contributed to the tetraploid diversity. Stift *et al*. [78] used crossing experiments to show that there are limited reproductive barriers between *R. amphibia *and the sympatric tetraploid *R. sylvestris *(L.) Besser, and concluded that gene flow between the two tetraploids is the probable reason for the high diversity found in tetraploid *R. amphibia *by Luttikhuizen *et al*. [23]. Kloda *et al*. [79] studied gene flow among diploid *Ononis *(*O. spinosa *L. and *O. intermedia *C.A.Mey. ex. A.K.Becker) and tetraploids (*O. repens *L. and *O. maritima *Dumort.) using microsatellites, and found that there were restrictions to gene flow between, but not within the ploidal levels. In the genus *Paeonia *L. homoploid hybrid species have been derived from allotetraploids, but not from the diploid progenitors, suggesting that chromosomal structural differences induced by polyploidy might create new opportunities for interspecific gene flow [80]. In line with this previous evidence, our study suggests that polyploids might tolerate introgression better than their diploid progenitors, as suggested by de Wet & Harlan [75] and Harlan & de Wet [76].

### Interploidal gene flow

In general, high genetic diversity in polyploids, as our network and genetic diversity analyses indicated for both *arenosa *and *lyrata*, can be explained by multiple independent origins of the polyploids, continuing introgression from the diploids, introgression from other polyploid species (see above), or as a result of subsequent evolution following one or more old polyploidisation events [see e.g., [3,6]]. Our network analyses for each species show no apparent clustering according to ploidal levels; i.e. we do not see a separation of diploids and tetraploids. For *arenosa *our IM analyses suggest that the diploids and the tetraploids are two distinct groups, with some migration from the diploid to the tetraploid level subsequent to the separation of the two gene pools. As the diploids from the Carpathians and the tetraploids from the Eastern Austrian Forealps and Germany are no longer sympatric, the migration we observe is probably the result of recurrent origin/introgression in the past. For *lyrata *our IM analysis suggests that the gene flow is bidirectional. This is also consistent with earlier studies [77] considering a broad geographic and population sampling that demonstrate a deeper evolutionary split between *arenosa *and *lyrata *irrespective of ploidal level variation.

In our flow cytometry data, we find signs of mixed-ploidy populations: a triploid plant was detected in the mainly diploid l2_CZE population of *lyrata *from the Czech Republic, and a diploid plant was detected in the mainly tetraploid a4_AUT1 population of *arenosa *from the Eastern Austrian Foreland. Also, the Central European *Arabidopsis *populations often have restricted distributions, and populations of different ploidal levels are sometimes only a few kilometres apart. Thus, introgression is possible, especially along disturbed sites, e.g., along roads. However, the generally low migration rates suggest that introgression remains a rare event. If this is the case, the small genus *Arabidopsis *contains polyploids with very different evolutionary fates when it comes to introgression. The allotetraploid *A. suecica *(Fr.) Norrl. ex O.E.Schulz has very low genetic diversity and has probably originated only once [34,81]. The allotetraploid *A. kamchatica *(Fisch. ex DC.) K.Shimizu & Kudoh has probably originated multiple times, and additionally experienced some later introgression from the parental diploids [14,35,82,83]. The tetraploid *arenosa *shows some signs of introgression, and the tetraploid *lyrata *shows signs of extensive ongoing introgression [cf. [45]]. As such, the genus *Arabidopsis *might be a good model system to use for developing the criteria and methods called for by Soltis *et al*. [6] for distinguishing between recurrent formation and introgression from progenitors as sources of diversity in polyploids. In this study we used IM analysis [67] in an attempt to make this distinction. As we were not able to obtain credible estimates of time divergence in our analyses, we could however not answer this question properly. Nevertheless, the results provide indications that different populations of the two species, *arenosa *and *lyrata*, are placed in different positions along the gradient of possible polyploid evolution models ranging from single event polyploidisation with subsequent reproductive isolation at one end of the gradient to polyploidisation with ongoing gene flow or recurrent polyploidisation at the other end.

Gene flow from diploids to tetraploid derivatives has long been acknowledged as relatively common [see e.g., [2,3,6] and references therein, [12]]. The question of gene flow in the opposite direction is more controversial. Stebbins [84] states that interploidal gene flow is usually unidirectional from diploids to tetraploids for two reasons: 1) offspring of triploid hybrids are usually tetraploid or close to it in chromosome number, and 2) diploids and tetraploids are often so highly incompatible that triploid offspring cannot be formed at all. However, studies involving natural triploids in euploid hybrid swarms show that triploids may produce 1*x*, 2*x*, and 3*x *gametes, and may therefore contribute to gene flow in both directions [19,20,85]. Indeed, gene flow from tetraploids to diploids has been observed in some taxa, e.g. *Dactylorhiza maculata *(L.) Soó [86] and *Betula *L [87]. In our study, we did not observe gene flow from tetraploid to diploid *arenosa *(*m *= 0; Figure 5), but as the different ploidal levels are allopatric, we cannot distinguish between genetic/genomic and geographic barriers. However, for *lyrata*, with diploids and polyploids in close proximity, gene flow seems to be bidirectional.

## Conclusions

In this study, we looked at the effect of polyploidisation on interspecific introgression, and interploidal gene flow using Central European *Arabidopsis *as a model system. There was no evidence for interspecific gene flow between 2*x arenosa *and 2*x lyrata*, which can be considered as good biological entities, but some support for gene flow into 4*x lyrata *and possibly 4*x arenosa*. Thus, whole genome duplication might decrease vulnerability to interspecific hybridisation and buffer negative effects of introgression. Interploidal gene flow was detected from 2*x *to 4*x *in both species, and from 4*x *to 2*x *in *lyrata*. For *arenosa*, the two ploidal levels are allopatric, and the lower level of gene flow could be the result of geographic as well as genetic barriers. In *lyrata*, however, where geographic barriers are limited, gene flow is bidirectional.

## Authors' contributions

MHJ carried out the molecular work and statistical analyses, and drafted the manuscript. DE carried out the isolation with migration analyses and helped to draft the manuscript. RS and MAK contributed to the sampling design, provided the samples and contributed to draft the manuscript. AKB was project leader, contributed in the molecular work, and drafted the manuscript. All authors read and approved the final manuscript.

## Supplementary Material

Additional file 1 **Table S1**. Dataset summary with primers, GenBank numbers, alignment lengths and minimum number of recombination events per region. Click here for file

Additional file 2 **Table S2**. Isolation with migration (IM) analyses: description of datasets. Click here for file

Additional file 3 **Table S3**. Isolation with migration analyses: mutation rates per fragment per generation. Click here for file

Additional file 4 **Table S4**. Signs of introgression among tetraploid *Arabidopsis arenosa *and *A. lyrata*. Click here for file

Additional file 5 **Table S5**. Diversity indices. Click here for file

Additional file 6 **Figure S1**. Isolation with migration analyses: effective population sizes and estimates of time for the def datasets. Click here for file

Additional file 7 **Figure S2**. Isolation with migration analyses of the seq datasets. Click here for file
