# Supplementary material for: Interspecific and interploidal gene flow in Central European Arabidopsis (Brassicaceae)
Source: BMC Evol Biol. 2011 Nov 29;11:346. doi: 10.1186/1471-2148-11-346 (PMC3247304; doi:10.1186/1471-2148-11-346)
Supplement: Additional file 1 — Table S1. Dataset summary with primers, GenBank numbers, alignment lengths and minimum number of recombination events per region. [file 1471-2148-11-346-S1.PDF]

Additional file 1: Table S1. Primers and annealing temperatures used for PCR prior to cloning and sequencing. CHS primers are taken from Koch et al. [1], *sc*ADH primers from Wright et al. [2], and *trn*L-F primers from Taberlet et al. [3]. Length gives the length of the final alignments. # seq gives number of sequences included in the final alignments. R_M gives minimal number of recombination events for each region [4].

Region	GenBank no.	Primer names	Primer sequences (5'-3')	Length /bp	# seq	R_M
CHS	GQ386503-	CHS-FOR1	CTTCATCTGCCCGTCCATCTAACC	1452	86	16
	GQ386588	CHS-REV5	GGAACGCTGTGCAAGAC			
*sc*ADH	GQ386589-	*sc*ADH-F	GGCATTCCTCCAGCGAC	1626	67	24
	GQ386654	*sc*ADH-R	CTTCCGTCGTCGTCTCTTC			
*trn*L-F	GQ386471-	c	CGAAATCGGTAGACGCTACG	700	32	1
	GQ386502	f	ATTTGAACTGGTGACACGAG			

1. Koch MA, Haubold B, Mitchell-Olds T: **Comparative evolutionary analysis of chalcone synthase and alcohol dehydrogenase loci in *Arabidopsis*, *Arabis*, and related genera (Brassicaceae)**. *Molecular Biology and Evolution* 2000, **17**(10):1483-1498.
2. Wright SI, Lauga B, Charlesworth D: **Subdivision and haplotype structure in natural populations of *Arabidopsis lyrata***. *Molecular Ecology* 2003, **12**:1247-1263.
3. Taberlet P, Gielly L, Pautou G, Bouvet J: **Universal primers for amplification of three non-coding regions of chloroplast DNA**. *Plant Molecular Biology* 1991, **17**:1105-1109.
4. Hudson RR, Kaplan NL: **Statistical properties of the number of recombination events in the history of a sample of DNA sequences**. *Genetics* 1985, **111**:147-164.
