# Supplementary material for: Interspecific and interploidal gene flow in Central European Arabidopsis (Brassicaceae)
Source: BMC Evol Biol. 2011 Nov 29;11:346. doi: 10.1186/1471-2148-11-346 (PMC3247304; doi:10.1186/1471-2148-11-346)
Supplement: Additional file 2 — Table S2. Isolation with migration (IM) analyses: description of datasets. [file 1471-2148-11-346-S2.PDF]

Additional file 2: Table S2. Isolation with migration (IM) analyses: reduction of datasets to nonrecombining blocks using IMgc. Fasta file indicates which dataset is included: all - including both taxa and ploidal levels, are - including *A. arenosa* only, lyr - including *A. lyrata* only, 2x - including diploids only. See text for explanation of D1-D22. Recombination gives Hudson's recombination parameter R [1] and minimal number of recombination events R_M [2]. We created two different kinds of IM input files through IMgc by choosing different prioritising parameters (pp): def prioritises number of sequences (pp = 1), whereas seq prioritises length of sequences (pp = 0.5). Start and stop gives the positions of the recombining blocks in the original alignments. The final number of sequences in each block is split into diploid and tetraploid *A. arenosa* (a2 and a4), and diploid and tetraploid *A. lyrata* (l2 and l4).

IMgc input					IMa2 input								
Fasta file	Region length/bp	# sequences	Recombination		IM input file	Start	Stop	Block length/bp	# sequences				Comment
			R	R_M					a2	a4	l2	l4	
all-CHS-D1.fas	1452	100	21.3	16	all-CHS-D1-def	263	677	414	10	31	14	29	
					all-CHS-D1-seq	203	896	693	3	15	14	24	
all-CHS-D2.fas			22.8	16	all-CHS-D2-def	263	701	438	10	35	14	29	
					all-CHS-D2-seq	203	896	693	6	17	14	24	
all-CHS-D3.fas			22.5	16	all-CHS-D3-def	263	701	438	9	33	14	28	
					all-CHS-D3-seq	215	953	738	2	18	14	24	too little a2
all-CHS-D22.fas			22.9	16	all-CHS-D22-def	263	776	513	8	32	14	27	
					all-CHS-D22-seq	263	990	727	1	13	14	26	too little a2
are-CHS-D1.fas	1452		39	13	are-CHS-D1-def	215	701	486	10	35			
					are-CHS-D1-seq	215	761	546	8	33			
are-CHS-D2.fas					are-CHS-D2-def	215	701	486	10	36			
					are-CHS-D2-seq	215	776	561	8	34			

are-CHS-D3.fas					are-CHS-D3-def	215	701	486	9	34		
					are-CHS-D3-seq	215	776	561	7	31		
are-CHS-D22.fas					are-CHS-D22-def	215	701	486	10	35		
					are-CHS-D22-seq	215	776	561	8	34		
lyr-CHS-D1.fas	1452		2	10	lyr-CHS-D1-def	423	1452	1029			16	25
					lyr-CHS-D1-seq	215	1452	1237			14	25
lyr-CHS-D2.fas					lyr-CHS-D2-def	423	1452	1029			16	25
					lyr-CHS-D2-seq	215	1452	1237			14	25
lyr-CHS-D3.fas					lyr-CHS-D3-def	423	1452	1029			16	25
					lyr-CHS-D3-seq	215	1452	1237			14	25
lyr-CHS-D22.fas					lyr-CHS-D22-def	215	1452	1237			14	26
					lyr-CHS-D22-seq	162	1452	1290			14	25
all-scADH-D1.fas	1626		70.05	24	all-scADH-D1-def	23	230	207	8	25	16	28
					all-scADH-D1-seq	527	806	279	5	21	14	30
all-scADH-D2.fas					all-scADH-D2-def	565	755	190	5	25	16	29
					all-scADH-D2-seq	527	806	279	5	22	14	29
all-scADH-D3.fas					all-scADH-D3-def	454	659	205	7	28	14	26
					all-scADH-D3-seq	527	806	279	5	22	12	29
all-scADH-D22.fas					all-scADH-D22-def	565	755	190	5	25	14	30
					all-scADH-D22-seq	565	818	253	4	21	14	30
are-scADH-D1.fas	1626	50	36.6	16	are-scADH-D1-def	1044	1467	423	8	32		
					are-scADH-D1-seq	1044	1626	582	8	28		
are-scADH-D2.fas			34.3	16	are-scADH-D2-def	1129	1626	497	10	38		
					are-scADH-D2-seq	849	1463	614	8	25		
are-scADH-D3.fas					are-scADH-D3-def	1129	1626	497	10	39		
					are-scADH-D3-seq	1010	1606	596	8	22		
are-scADH-D22.fas					are-scADH-D22-def	1129	1626	497	10	38		
					are-scADH-D22-seq	1010	1463	453	8	26		
lyr-scADH-D1.fas	1626	48	37.7	16	lyr-scADH-D1-def	353	704	351			14	28
					lyr-scADH-D1-seq	565	1010	445			11	24

lyr-scADH-D2.fas				lyr-scADH-D2-def	353	755	402		16	28	
				lyr-scADH-D2seq	565	1028	463		11	22	
lyr-scADH-D3.fas				lyr-scADH-D3-def	565	1010	445		11	28	
				lyr-scADH-D3-seq	556	1109	553		9	27	
lyr-scADH-D22.fas		33.5	16	lyr-scADH-D22-def	565	1010	445		11	29	
				lyr-scADH-D22-seq	556	1109	553		9	27	
2x-CHG.fas				2x-CHS-D1-def	445	953	508	7	16		
				2x-CHS-D1-seq	395	953	558	5	14		
2x-scADH.fas	1626			2x-scADH-D1-def	1130	1455	325	9	14		
				2x-scADH-D1-seq	181	1156	975	0	11		too little a2

1. Hudson RR: **Estimating the recombination parameter of a finite population model without selection**. *Genetical Research* 1987, **50**:245-250.
2. Hudson RR, Kaplan NL: **Statistical properties of the number of recombination events in the history of a sample of DNA sequences**. *Genetics* 1985, **111**:147-164.
