# Supplementary material for: Interspecific and interploidal gene flow in Central European Arabidopsis (Brassicaceae)
Source: BMC Evol Biol. 2011 Nov 29;11:346. doi: 10.1186/1471-2148-11-346 (PMC3247304; doi:10.1186/1471-2148-11-346)
Supplement: Additional file 3 — Table S3. Isolation with migration analyses: mutation rates per fragment per generation. [file 1471-2148-11-346-S3.PDF]

Additional file 3: Table S3. Isolation with migration analyses: mutation rates per fragment per generation. The rates are based on a per site mutation rate of $1.075 \cdot 10^{-8}$ scaled by the length of each fragment following Slotte et al. [1].

Region	Mutation rate
CHS	$2.13 \cdot 10^{-5}$
*sc*ADH	$1.55 \cdot 10^{-5}$
*trn*L-F	$5.30 \cdot 10^{-6}$
Mean	$1.21 \cdot 10^{-5}$

1. Slotte T, Huang H, Lascoux M, Ceplitis A: **Polyploid speciation did not confer instant reproductive isolation in *Capsella* (Brassicaceae)**. *Molecular Biology and Evolution* 2008, **25**(7):1472-1481.
