# Supplementary material for: Interspecific and interploidal gene flow in Central European Arabidopsis (Brassicaceae)
Source: BMC Evol Biol. 2011 Nov 29;11:346. doi: 10.1186/1471-2148-11-346 (PMC3247304; doi:10.1186/1471-2148-11-346)
Supplement: Additional file 4 — Table S4. Signs of introgression among tetraploid Arabidopsis arenosa and A. lyrata. [file 1471-2148-11-346-S4.PDF]

Additional file 4: Table S4. Signs of introgression among tetraploid *Arabidopsis arenosa* and *A. lyrata*. are-like and lyr-like give number of alleles clustering with *A. arenosa* and *A. lyrata*, respectively, for each of the two low-copy nuclear regions (CHS and *sc*ADH).

Taxon	Specimen	CHS are-like	CHS lyr-like	*sc*ADH are-like	*sc*ADH lyr-like
A. arenosa	a4_AUT1_1	3	3	3	-
	a4_AUT1_2	2	1	2	1
	a4_AUT2_17	2	2	3	-
	a4_AUT2_18	3	1	2	-
	a4_AUT4_14	3	1	3	-
	a4_AUT4_15	3	1	3	-
	a4_GER_1	1	1	2	-
A. lyrata	l4_AUT1_11	1	2	1	1
	l4_AUT1_12	3	1	-	2
	l4_AUT4_2	1	3	1	1
