# Supplementary material for: Interspecific and interploidal gene flow in Central European Arabidopsis (Brassicaceae)
Source: BMC Evol Biol. 2011 Nov 29;11:346. doi: 10.1186/1471-2148-11-346 (PMC3247304; doi:10.1186/1471-2148-11-346)
Supplement: Additional file 5 — Table S5. Diversity indices. [file 1471-2148-11-346-S5.PDF]

Additional file 5: Table S5. Diversity indices calculated for the different regions and the different data subsets: *are* 2*x* and *lyr* 2*x* are diploid *arenosa* and *lyrata*, respectively, *are* 4*x* D1-D22 and *lyr* 4*x* D1-D22 are tetraploid subsets created through different sequence duplication schemes, and *are* 4*x* and *lyr* 4*x* are the means of these subsets (CHS and *sc*ADH), or the unduplicated tetraploid subsets (*trn*L-F). # seq gives the number of sequences, Length gives sequence lengths excluding gap sites, S gives number of segregating sites, # hapl gives number of haplotypes, Hd ± sd gives gene diversity ± standard deviation, π gives nucleotide diversity, k gives average number of nucleotide differences.

		are 2x	are 4x D1	are 4x D2	are 4x D3	are 4x D22	are 4x	lyr 2x	lyr 4x D1	lyr 4x D2	lyr 4x D3	lyr 4x D22	lyr 4x
CHS	# seq	12	40	40	40	40	40	16	32	32	32	32	32
	Length	1417	1422	1422	1422	1422	1422	1421	1422	1422	1422	1422	1422
	S	43	76	76	76	76	76	25	56	56	56	56	56
	# hapl	11	28	28	28	28	28	10	17	17	17	17	17
	Hd ± sd	0.98 ± 0.04	0.98 ± 0.01	0.98 ± 0.01	0.98 ± 0.01	0.98 ± 0.01	0.98	0.93 ± 0.04	0.97 ± 0.01	0.97 ± 0.01	0.97 ± 0.01	0.97 ± 0.01	0.97
	π ± sd	0.011 ± 0.006	0.015 ± 0.008	0.014 ± 0.007	0.015 ± 0.007	0.014 ± 0.007	0.015	0.010 ± 0.005	0.012 ± 0.006	0.012 ± 0.006	0.012 ± 0.006	0.012 ± 0.006	0.012
	k ± sd	15.61 ± 7.50	21.38 ± 9.63	20.71 ± 9.34	20.82 ± 9.40	20.70 ± 9.34	20.90	13.77 ± 6.53	16.62 ± 7.60	17.24 ± 7.87	16.91 ± 7.72	16.46 ± 7.53	16.81
*sc*ADH	# seq	10	40	40	40	40	40	16	32	32	32	32	32
	Length	1133	1035	1035	1035	1035	1035	1305	1188	1188	1188	1188	1188
	S	104	144	144	144	144	144	66	94	94	94	94	94
	# hapl	9	24	24	24	24	24	9	17	17	17	17	17
	Hd ± sd	0.98 ± 0.05	0.97 ± 0.01	0.97 ± 0.01	0.97 ± 0.01	0.98 ± 0.01	0.97	0.94 ± 0.03	0.96 ± 0.02	0.95 ± 0.02	0.95 ± 0.02	0.96 ± 0.01	0.96
	π ± sd	0.113 ± 0.060	0.102 ± 0.050	0.100 ± 0.048	0.109 ± 0.053	0.111 ± 0.055	0.105	0.051 ± 0.026	0.071 ± 0.035	0.070 ± 0.034	0.070 ± 0.034	0.066 ± 0.034	0.069
	k ± sd	179.28 ± 84.07	165.48 ± 72.39	161.32 ± 70.58	176.52 ± 77.20	178.97 ± 78.27	170.57	72.11 ± 32.82	99.84 ± 44.04	98.62 ± 43.51	98.28 ± 42.36	93.52 ± 41.24	97.57
*trn*L-F	# seq	6					10	8					8
	Length	688					688	689					688
	S	2					3	4					3

# hapl	3	3	3	3
Hd ± sd	0.6	0.82	0.71	0.86
	± 0.22	± 0.07	± 0.12	± 0.11
π ± sd	0.006	0.005	0.009	0.008
	± 0.004	± 0.003	± 0.005	± 0.005
k ± sd	4.07	3.76	6.14	5.57
	± 2.36	± 2.07	± 3.27	± 2.99
