# Supplementary material for: Interspecific and interploidal gene flow in Central European Arabidopsis (Brassicaceae)
Source: BMC Evol Biol. 2011 Nov 29;11:346. doi: 10.1186/1471-2148-11-346 (PMC3247304; doi:10.1186/1471-2148-11-346)

Additional file 6: Figure S1. Isolation with migration analyses of the def datasets (Table S2). (a,b) effective population sizes ($q = 4N\mu$) for *A. arenosa* and *A. lyrata*, respectively. (b,d) time since population split (t) for the same datasets.

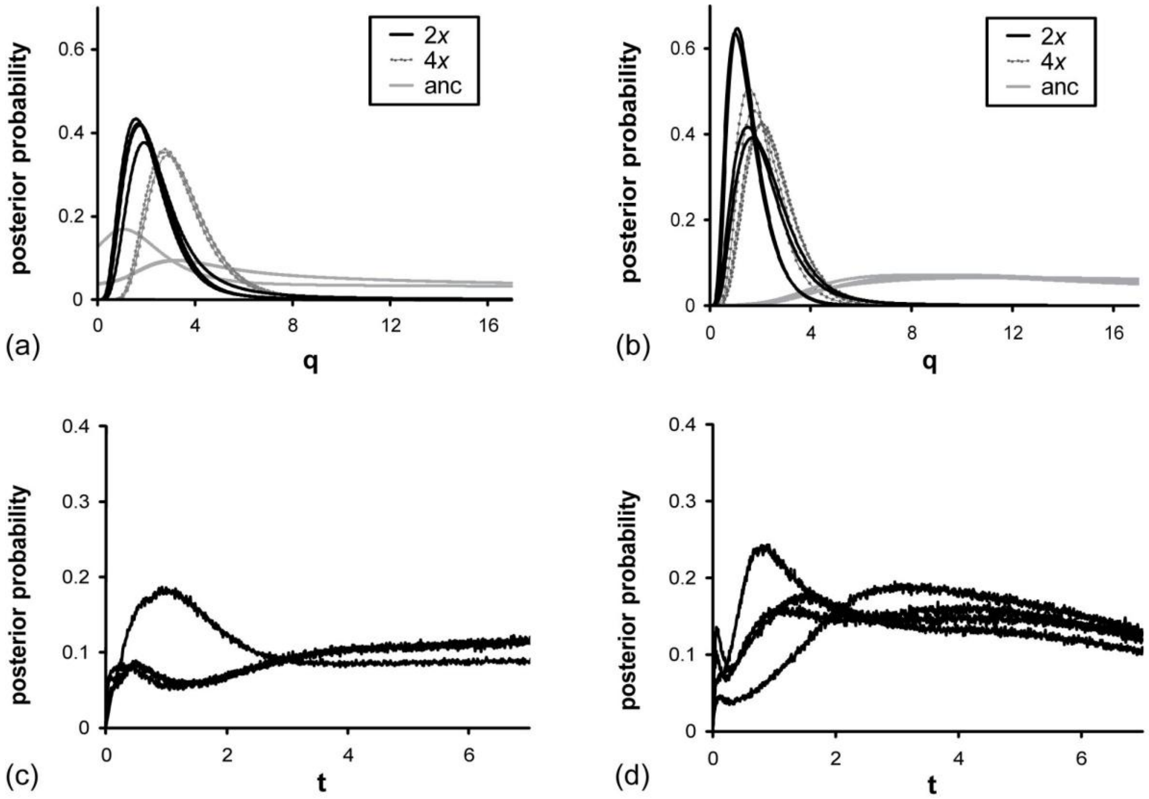

Supplement: Additional file 6 — Figure S1. Isolation with migration analyses: effective population sizes and estimates of time for the def datasets. [file 1471-2148-11-346-S6.PDF]
