# Supplementary material for: Interspecific and interploidal gene flow in Central European Arabidopsis (Brassicaceae)
Source: BMC Evol Biol. 2011 Nov 29;11:346. doi: 10.1186/1471-2148-11-346 (PMC3247304; doi:10.1186/1471-2148-11-346)

Additional file 7: Figure S2. Isolation with migration analyses of the seq datasets (Table S2). (a, b) migration rates (*m*), (c, d) effective population sizes ($q = 4N\mu$), and (e, f) time since population split (*t*) for *A. arenosa* (a, c, e) and *A. lyrata* (b, d, f).

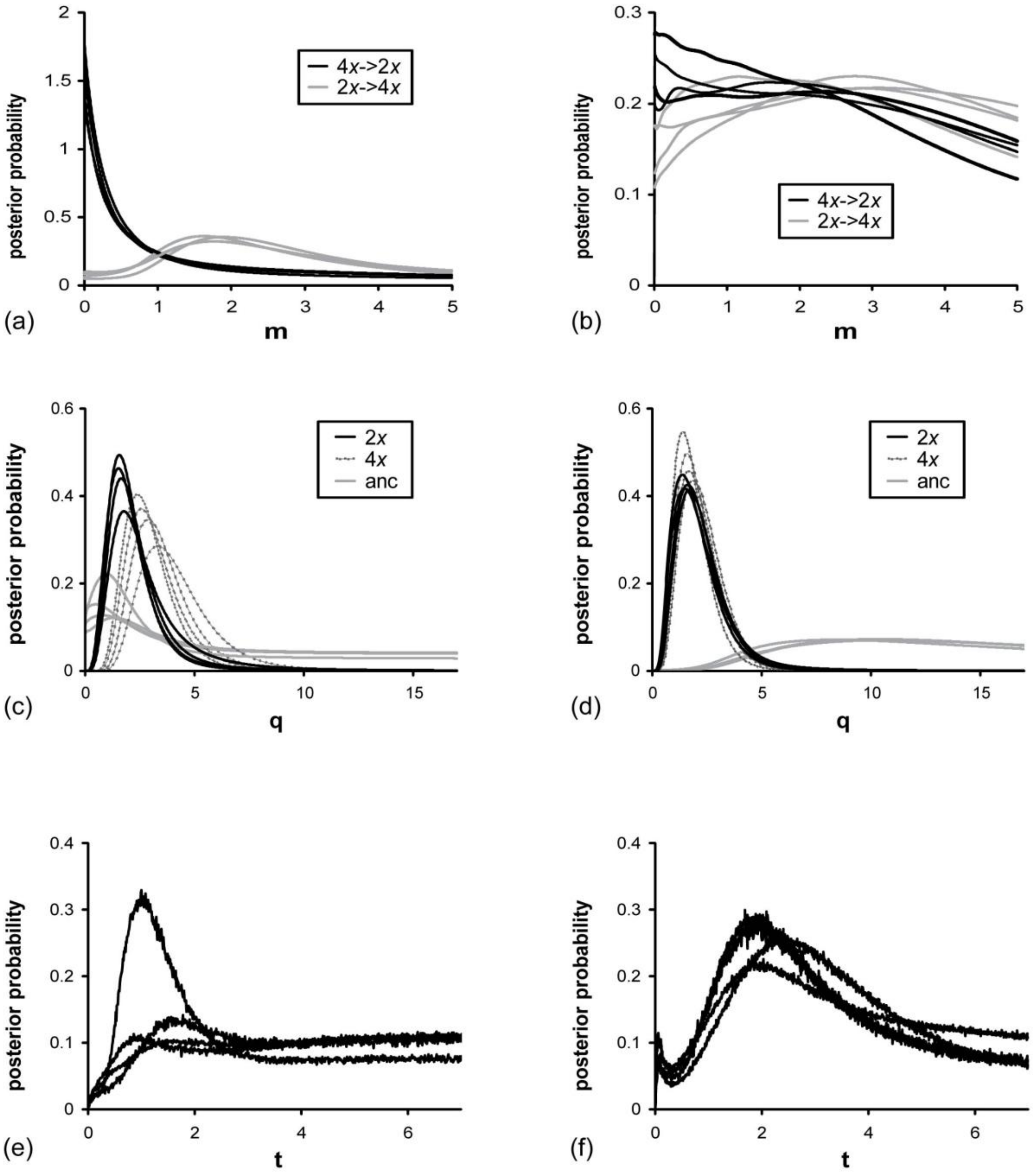

Supplement: Additional file 7 — Figure S2. Isolation with migration analyses of the seq datasets. [file 1471-2148-11-346-S7.PDF]
